# The impact of shift work on paramedics and their practice: Protocol for a simulated paramedic shift work study

**DOI:** 10.1371/journal.pone.0319569

**Published:** 2025-03-26

**Authors:** Laura M. Hirello, Sean P. A. Drummond, Kelly-Ann Bowles, Alexander P. Wolkow

**Affiliations:** 1 School of Psychological Sciences, Monash University, Clayton, Victoria, Australia; 2 Department of Paramedicine, Monash University, Frankston, Victoria, Australia; PLOS: Public Library of Science, UNITED KINGDOM OF GREAT BRITAIN AND NORTHERN IRELAND

## Abstract

Paramedics make up an integral part of modern healthcare systems, however, there remains a paucity of research on the occupational demands of their role. The majority of paramedics in Australia work on a rotating shift schedule. Despite the documented impact of shift work on sleep loss, and resultant performance and physiological impairments, few studies have examined the implications of shift work in paramedic populations. This study explores the impact of shift work, and the resultant circadian rhythm disruption, on paramedic decision making, work performance and underlying physiology. The study aims to recruit 22 Australian paramedics with an entry to practice scope. In pairs, participants complete a two 12-hour day shift, two 12-hour night shift simulated work rotation. All sleep opportunities during the rotation occur in the Monash Sleep and Circadian Medicine Laboratory and are monitored with polysomnography. Simulated paramedic shifts take place in the Monash Paramedic Simulation Centre, where participants engage in high-fidelity immersive paramedic scenarios throughout the shift. Paramedic scenarios are recorded for asynchronous evaluation by subject matter experts. In addition to paramedic scenarios, participants complete two cognitive and decision-making batteries during each shift. Biological markers are also collected throughout the rotation to assess changes in paramedics’ stress responses (i.e., alpha-amylase, cortisol, heart rate variability, cytokines), as well as circadian phase (i.e., 6-sulfatoxymelatonin). The novel simulated work environment study design contributes significantly to the paramedic body of literature through advancing our understanding of the impacts of shift work on paramedics. This study provides valuable insights into the nature of paramedic work and generates future research directions that will allow for further examination and understanding of the occupational demands of the paramedic profession.

## Introduction

In recent decades, Australian paramedics have solidified their role and importance in the healthcare sector [1,2]. Paramedics in Australia require a bachelor’s degree in paramedicine and are regulated at the national level. Despite their integral role in modern healthcare systems, paramedic research lags behind analogous healthcare professions [3]. This is especially true for research focused on the occupational conditions and experiences of paramedics. Limited research has examined occupational demands of the role, including shift work and sleep loss, and their impact on individuals engaging in paramedic work. As the public becomes aware of the challenges and pressure paramedics experience, there is an increased call to examine these workplace stressors and their impact on performance [4]. Further, high turnover rates among paramedics strengthens the need for information about the role of workplace stressors on occupational health [5–7]. Greater work in this area will not only shed light on paramedic occupational experience but give vital information about how to make the job safer and more sustainable moving forward.

Paramedics operate in 24/7 conditions. The paramedic profession, and greater healthcare system, is built on the concept of round the clock availability of medical care [1]. This results in paramedics engaging in shift work, often providing high acuity medical care during the biological night. The majority of paramedics (92%) in Australia are employed by jurisdictional ground ambulance services, with schedules including rotating shifts [6]. Rotating shift schedules most often involve a combination of day and night shifts occurring over consecutive days, followed by a short period of time off. Shift work, in general, is known to result in sleep loss and circadian rhythm disruption [8,9]. However, there is a paucity of research into the specific effects of shift work in the paramedic population [10,11]. Thus, it remains unclear how, and to what degree, shift work and the resultant sleep loss and circadian disruption impacts paramedics, their work performance, decision making, and underlying stress physiology.

Accurate and consistent measures of paramedic work performance are challenging to objectively assess in real-world conditions. The nature of paramedic work involves high stress situations requiring complex clinical decision making [1]. The paramedic work environment is often unpredictable and highly variable, with each call for service resulting in a different combination of medical and non-medical elements to consider. Within these complex and dynamic situations, paramedic work performance significantly impacts patient outcomes [12] and often shapes patient trajectory and experience within the healthcare system [2,13–15]. However, studying the effects of these stressors on real-life paramedic work performance is unsafe and impractical. Given these challenges, simulator-based work environments create a powerful model to understand the effects of real-world stressors on work performance.

Simulation based assessment (SBA) is a common practice in paramedicine and the greater medical field. Simulated work environments provide a validated and realistic setting for SBA in paramedic education [16]. These environments allow for creation of novel real-world situations that include details and information impacting paramedic decision making, while maintaining consistency in how a situation is facilitated [17]. Using existing qualitative models of out-of-hospital decision making and internationally validated paramedic competency scales, simulated work environments and SBAs can be constructed to both align with reality while simultaneously highlighting specific aspects of paramedic performance [17–19]. Paramedic work domains like situational awareness, clinical decision making, and information gathering are particularly well suited to evaluation in SBA as they rely on details included in the simulated work environment [20]. Engaging in SBA throughout a shift work rotation allows for exploration of how multiple elements of judgement and cognition impact paramedics’ overall work performance in response to shift work and related sleep loss.

Decision making is one of the dimensions underpinning overall paramedic work performance [19]. Recently, clinical decision making has become an area of interest for all types of healthcare providers, paramedics included [21,22]. However, most prior research has relied on *ex post facto* self-reflection or surveys. Few studies have quantitatively measured or experimentally tested decision-making strategies of paramedics, particularly as they progress through a multi-day shift work rotation [23]. Here, we will collect multiple measures of operational and experimental decision making throughout 4 days of simulated shift work.

Shift work is known to impact a range of physiological responses. Even during day shifts, the demands of paramedic work are shown to impact stress response systems, including the sympathetic nervous system and hypothalamic-pituitary-adrenal (HPA)-axis [24]. Research in other healthcare professionals has demonstrated a single overnight shift impacts immune system functioning and causes dysregulation of inflammatory stress response markers [25]. Melatonin is a hormone associated with the sleep-wake cycle of the circadian rhythm [26]. Shift work is known to disrupt melatonin levels in workers and is linked to health impairments [26, 27]. Despite the documented health risks associated with shift work, there is very limited research looking at physiological changes in paramedics as they progress through a shift work rotation. Here, we will collect biological markers of stress response and circadian rhythms throughout 4 days of simulated shift work.

One of the main differentiating features of paramedicine, as compared to other areas of healthcare, is the resource limited environment within which clinicians operate. Environmental resource scarcity increases the reliance on paramedic partnerships, teamwork and communication. Communication is identified in both the paramedic research and policy literature as a skill fundamental to the profession [28,29]. Paramedic communication, not only with other paramedics and healthcare providers, but with patients and families, is essential for effective information gathering, establishing rapport, and building trust required for high quality patient care [30]. Research demonstrates sleep deprivation can impair communication and language comprehension [31,32]. Further, preliminary work has demonstrated the complexity of auditory language can modulate small team performance during night shifts [32]. However, no work has examined communication in paramedic teams as they progress through a shift work rotation. Here, we will collect both written and verbal communication data throughout 4 days of simulated shift work.

## Aims and hypotheses

The present study uses a simulated shift work protocol to assess how multiple days of shift work, and the resultant circadian rhythm disruption and sleep restriction, impact paramedics and their occupational performance. The protocol involves paramedics, in teams of two, progressing through a two-day, two-night, simulated shift work rotation. During each simulated shift, participants complete simulated paramedic scenarios and validated tasks to assess clinical work performance and decision making, respectively. Participant performance during paramedic scenarios is recorded and their sleep quality and quantity is monitored during all sleep opportunities within the shift rotation. Finally, biological samples are collected from participants at key timepoints throughout the shift rotation to assess physiological stress responses and circadian rhythm disruption.

Aim 1: To examine the impact of simulated shift work, and the resultant sleep loss and circadian rhythm disruption, on paramedic decision making and work performance.

Hypothesis 1A: Overall participant work performance, as measured by a modified paramedic Global Rating Scale (GRS) focused on decision making, situational awareness, and communication dimensions, will be worse as sleep loss accumulates and subjective fatigue increases both over consecutive shifts (i.e., day one to day two), and throughout the rotation (i.e., day one to night two). Within individual participants, existing weaknesses in specific paramedic work dimensions will be exacerbated during periods of acute sleep loss.

Hypothesis 1B: Participants will shift to less cognitively taxing decision-making strategies, relying on reinforcement and representative heuristics, over consecutive shifts, and as the rotation progresses.

Hypothesis 1C: Participants will demonstrate increased risk seeking behaviours over consecutive shifts, and as the rotation progresses.

Aim 2: To examine physiological changes in paramedics as they progress through a simulated shift work rotation.

Hypothesis 2: Patterns of cortisol, alpha-amylase (sAA) and heart rate variability (HRV) will demonstrate increasing dysregulation in participants over consecutive shifts and as the shift rotation progresses.

Aim 3: To evaluate paramedic communication throughout the simulated shift rotation.

Hypothesis 3: Participant communication will degrade in effectiveness over consecutive shifts and as the shift rotation progresses, evidenced by changes in language tone, quantity, and clarity.

## Methods

### Study design

There are three phases of data collection in this study. The first is an online survey that establishes baseline sleep and health behaviours in participants. The second phase is a 7-day at-home sleep monitoring period. During this phase, participants are asked to maintain an 8- to 9-hour sleep opportunity each night, wear an actigraphy device (Actiwatch 2, Spectrum or Spectrum Pro, Philips Respironics, OR, USA) to monitor sleep, and complete a short online sleep diary each morning. The goal of the at home sleep monitoring is to normalize participants’ sleep duration prior to the final data collection phase, the simulated shift work rotation.

**Fig 1:**
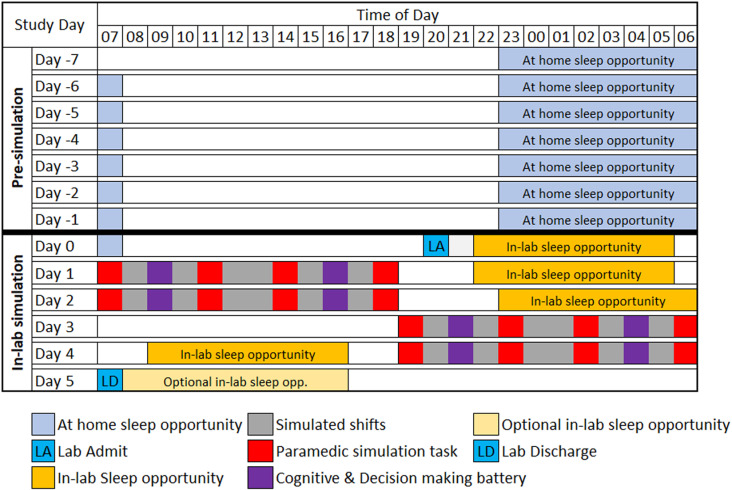
Seven day at-home sleep monitoring, and 5 day in-lab simulated shift rotation.

The simulated shift work rotation begins immediately following the 7 days of at home sleep monitoring (Fig 1). Participants complete the simulated shift work rotation in pairs, completing two 12-hour day shifts, followed by two 12-hour night shifts. All data collection will be conducted at the Monash University Sleep and Circadian Medicine Laboratory and the Monash Paramedic Simulation Centre. The study has received ethics approval from the Monash University Human Research Ethics Committee (Project ID: 31691).

Participants enter the Monash Sleep and Circadian Medicine Laboratory the evening before the first day shift of the simulated rotation (Day 0; Fig 1). Upon entering the laboratory, participants are familiarized with the equipment, tasks and simulation areas used during the study, and then have an 8-hour sleep opportunity. For all sleep opportunities in the laboratory, participants are set up with polysomnography to measure sleep quantity and sleep stages.

At the start of each shift, participants are brought to the Monash Paramedic Simulation Centre. During shifts, they complete a total of four paramedic scenarios using high-fidelity simulation equipment, and two decision-making and cognitive batteries as well as a teamwork task (Fig 1). At the end of each shift, participants return to the Monash Sleep and Circadian Medicine Laboratory, where they have a sleep opportunity. Participants are aware of the shift start and end times and determine their own sleep and wake times within a pre-set sleep opportunity window. Biological sampling, including urine, saliva, and blood, are conducted at regular intervals throughout the protocol, and HRV data is collected during each of the paramedic scenarios and decision-making and cognitive batteries. Participants are discharged from the study on the morning of Day 5, following completion of their last night shift (Fig 1). For safety purposes, all participants are given the option of either commuting home via taxi or having a safety sleep in the laboratory immediately following study discharge.

### Participants and recruitment

Inclusion criteria include paramedics registered with the Australian regulatory body (i.e., Australian Health Practitioner Regulation Agency) practicing at the Advanced Life Support (or equivalent entry-to-practice) level in Australia. Participants must also possess 1 to 10 years of paramedic experience and currently work a rotating shift schedule (i.e., a combination of day and night shifts) in Australia. Participants are recruited using advertisements distributed via peak professional bodies and unions representing Australian paramedics. Study advertisements are also circulated on social media, and eligible participants from previous studies are sent invitations to participate. The recruitment target is 22 participants, giving power of 0.8 with alpha equal to .05 to detect a medium effect of 0.3 for work performance and decision-making outcomes. Participants will be screened to ensure they meet eligibility criteria via a phone call, during which written informed consent will be obtained via a participant-completed online form.

### Polysomnography

The polysomnography set up for each sleep opportunity utilizes a 6-channel electroencephalogram (EEG) montage with electrodes F3/F4, C3/C4 and O3/O4. Eye movement and chin muscle activity is monitored through electrooculogram (EOG) and electromyogram (EMG) respectively, with electrocardiogram (ECG) and pulse oximetry included to assist in sleep staging. Polysomnography during the first sleep opportunity (i.e., between Days 0 and 1; Fig 1) also includes measures of breathing, oximetry, snoring, and limb movement to screen for obstructive sleep apnea and periodic limb movement disorder.

### Paramedic scenarios

Participants complete four simulated paramedic scenarios across the span of each shift (Fig 1). As participants complete the study in pairs, they work with the same partner for the duration of the full shift rotation. During every shift, each participant acts as the ‘attending paramedic’ in two (of the four) scenarios, while the other participant acts as the ‘partner paramedic’. Participants switch off the attendant role after each scenario. As there are four scenarios per shift, participants always act as the attending paramedic in the same order (i.e., if a participant attends the first scenario of shift one, they will attend the first scenario of all subsequent shifts).

The paramedic scenarios completed within every shift are unique. Each scenario fits into one of four clusters of differential diagnoses, with a scenario from each cluster within each shift. The order of each unique scenario is counterbalanced across rotations. The four differential diagnosis clusters include presentations of: 1) chest pain; 2) shortness of breath in a young adult (15 – 40 years old); 3) trauma; and 4) shortness of breath in an older adult (65 + years old). Scenarios follow pre-set algorithms that determine patient condition, including vital signs, and decompensation or improvement. Algorithms are programmed into the high-fidelity cardiac monitors (REALITi360, iSimulate, Australia) to ensure consistent administration. The algorithms progress in response to a combination of paramedic actions (e.g., patient condition improves after appropriate administration of a medication) and pre-set timings (e.g., patient condition deteriorates 4 minutes after patient contact). The use of algorithms allows the scenarios to be applied consistently while remaining realistic and responsive to paramedic actions. The non-medical details of each patient and scenario (e.g., patient demographics, psychosocial circumstance, physical environment, bystanders etc.) are unique and vary from one scenario to the next. Medical details for calls within the same diagnostic cluster often look similar at initial patient presentation, with only slight differences based on the underlying issue. This simulation design requires participants to successfully differentiate between clinically similar patient presentations, allowing for greater sensitivity in the assessment of participant information gathering, clinical decision making, and communication. Variation and detail in the non-medical elements of the scenarios create greater contextualization of the medical presentation. Not only do non-medical details introduce variables that contribute to greater decision making complexity, they increase participant immersion into the scenario and validity of the simulation [20]. For example, in the first iteration of the shortness of breath in a young adult diagnostic cluster, the patient could be a 21-year-old male with asthma exacerbation, while in the second iteration, the patient could be a 17-year-old female in anaphylaxis. All scenarios were subjected to pilot testing with paramedic undergraduate students to ensure details were cogent and algorithms progressed appropriately.

**Fig 2:**
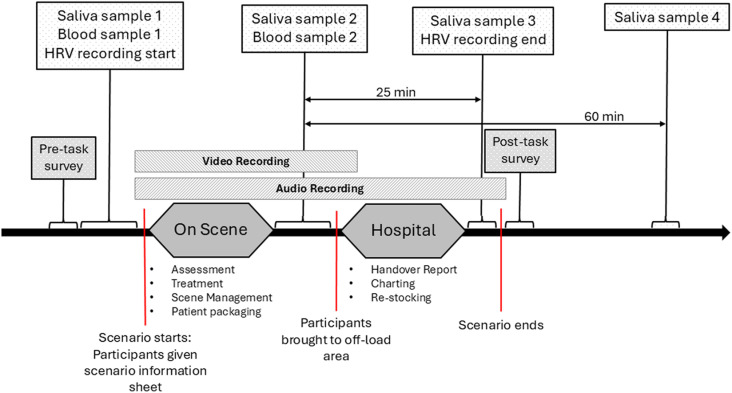
Scenario timeline. HRV =  Heart rate variability.

At the start of each paramedic scenario (Fig 2), participants are given preliminary information about the scenario (i.e., patient age, sex and chief complaint; response type; approximate response time), similar to what would be received from an ambulance dispatch centre. Prior to the start of the scenario, the first saliva sample is collected from the participants. They are then moved to mock ambulance seats where they are given the scenario dispatch information. The pair of participants have the chance to discuss the scenario information they have received and formulate a plan, similar to the reality of what paramedics do while driving to a call for service. When participants arrive ‘on scene’ of the emergency, they are led into the high-fidelity Monash Paramedic Simulation Centre which has projectors that allow for pre-recorded audio and video backgrounds to be shown on the surrounding walls, providing an immersive experience that assists in creating a setting analogous to reality.

Two researchers run the scenario, with one acting as the patient while the other facilitates (e.g., operates iREALITi360) and fills in any additional information the ‘patient’ is unable to provide. Most medical equipment and consumables used during the scenarios are the same as those used by ambulance services in Australia. Wherever possible, paramedic skills must be fully enacted by participants on the ‘patient’ before the facilitator will provide information about assessment findings or intervention response.

Paramedic scenarios are not time limited. Time ‘on scene’ runs as close to real time as possible. When the participants choose to leave scene, the patient must be moved to the stretcher and loaded into the mock ambulance located in the simulation laboratory. Upon ‘departing the scene’, the simulation is paused to allow for biological sampling (i.e., saliva, blood; Fig 2). Following collection of these samples, the simulation resumes and participants are told they have ‘arrived’ at the hospital (Fig 2). Participants must then unload the stretcher from the ambulance and bring the patient into a mock hospital space located a short distance from the simulation area. Importantly, the lights in the mock hospital space are on at full strength (>300 lux), as would be the case in a real hospital. In this space, participants must give a verbal patient handover and then have 20 minutes to complete a patient care report and restock equipment used during the scenario (Fig 2). Biological sampling will continue at key timepoints throughout the ‘hospital’ portion of the scenario. Once the 20-minute hospital period is complete, the scenario is deemed complete, however biological sampling continues until all samples in the series are collected (Fig 2).

### Decision-making and cognitive tasks

During each shift, participants complete a cognitive and decision-making battery (on a laptop) at two timepoints, spaced relatively evenly between the paramedic scenarios (Fig 1). The cognitive tasks include the 3-minute Psychomotor Vigilance Task (PVT) [33], a 2-back working memory task [34], and the Balloon Analogue Risk Task (BART) [35]. The decision-making portion of the battery immediately follows the cognitive portion and includes three variations of the Bayesian Decision Task (BDT), a standard version, heuristic variant and an emotional content variant [36], as well as the framing effect task [37]. To increase their efficacy, both the BART and framing effect task are monetarily incentivized based on participant performance [38].

The final task of the battery is a cooperative teamwork game called Keep Talking and Nobody Explodes (KTaNE) [39]. In KTaNE, one participant has a laptop in front of them with a bomb on the screen, while the other participant has the bomb diffusion manual. Participants are arranged so they cannot see what the other participant has in front of them. They have five minutes to defuse the bomb by working through a series of problem-solving modules. The KTaNE is completed twice per battery, with each participant taking a turn in each role. All communication throughout the KTaNE is recorded via a portable audio recorder (ICD-PX470, Sony, Japan).

### Biological sampling

Biological samples, including urine, saliva, and blood, are taken at multiple timepoints throughout the shift work rotation. Urine is collected for measurement of 6-sulfatoxymelatonin (aMT6s), a metabolite of melatonin. This marker is used to measure the participants’ circadian phase throughout the rotation. Collection of urine begins upon entry to the laboratory and continues throughout the rotation in 4-hour intervals during waking periods and approximately 8-hour intervals during sleeping periods.

Saliva samples are collected for measurement of stress markers, including sAA and cortisol. Samples for both markers are collected immediately before, after and at the post scenario + 25-minute timepoint for each simulated paramedic scenario (Fig 2). Saliva samples collected at the post scenario + 60-minute timepoint are used to assess cortisol (Fig 2). Blood samples are collected before and after the final paramedic scenario of each shift (Fig 2) for measurement of inflammatory stress markers, including pro- and anti-inflammatory cytokines. Finally, participants wear heart rate monitors (Polar V800, Polar, Finland) during each paramedic scenario and cognitive and decision-making battery. The monitors record continuous HRV data during, and for 30 minutes after, each scenario and testing battery.

### Primary outcomes

The primary outcomes of the study are changes in paramedic work performance, decision making, physiological stress responses, and communication during the simulated shift rotation. During each of the paramedic scenarios, work performance data is collected using the audio and video recording capabilities of the Monash Paramedic Simulation Centre. This centre has five ceiling mounted cameras that together provide a full view of the simulation space. Video streams from each camera, as well as the simulated patient monitor (i.e., iREALITi360) are recorded. Audio data is also collected during the scenarios via two wireless clip-on microphones (Wireless GO II, RODE, Australia) worn by each participant. The researcher acting as the ‘patient’ has an additional audio recorder to ensure the verbal hospital report is recorded when participants are outside of the simulation area. Each of these video and audio recordings will be circulated to a team of paramedic subject matter experts for evaluation of participant work performance during the scenarios. A modified version of the paramedic GRS [19] that focuses on the dimensions of situational awareness, decision making and communication will be used by subject matter experts to assess work performance. Evaluators will be blinded to the rotation and shift within which the scenario occurred. Full descriptions of the three dimensions being used for evaluation, as well as rating scale definitions will be provided to all evaluators. Individual paramedic work performance is hypothesized to degrade both over consecutive shifts and during simulated night shifts as compared to simulated day shifts. Degradation of performance is expected to be reflected through inefficient decision making, decreased situational awareness, and poor communication.

Results from the batteries of decision making and cognitive tasks (i.e., BDT, BART, framing effects task, PVT and 2-back working memory task) will be analysed to detect changes in decision-making strategy and risk preference. As the simulated work rotation progresses, we hypothesize participants will opt for intuition-based strategies utilizing heuristics over more cognitively taxing decision-making processes. Participant decisions are expected to be more risk seeking during consecutive shifts and as the rotation progresses. Change in decision making strategy is expected to be a moderating factor to the hypothesized degradation in work performance.

Physiological markers of stress will be examined in paramedics using the biological samples and heart rate data collected throughout the shift rotation. These measures will be analysed for stress markers (e.g., HRV, cortisol, sAA), which will be used to determine whether changes in sympathetic nervous system and HPA-axis activity occur in response to work performed during the paramedic scenarios. These biological markers will be compared across timepoints in the shift rotation to explore whether physiological stress responses are adversely impacted by the shift schedule. Additionally, participants’ circadian phase will be assessed (via aMT6s) to examine interactions between the shift rotation, circadian rhythm, and stress responses. Given the simulated night shifts include exposure to bright fluorescent lights and activity during the biological night, circadian rhythm disruption is expected. As there is an established association between menstrual cycle changes and circadian rhythm disruption [40–42], circadian phase is hypothesized to show increased disruption in all persons who menstruate.

The final primary outcome is communication, which will be assessed through analysis of multiple media. The narrative portion of the patient care report will be evaluated using linguistic inquiry and wordcount software (LIWC) [43]. Audio recordings of scenarios and KTaNE will be analysed using feature extraction software (openSMILE) to evaluate multiple speech related dimensions including pitch, voice quality and loudness [44]. As the rotation progresses, participants are expected to develop a familiarity with the communication style of their partner and start to build their own verbal shorthand. However, the effectiveness of familiar language is hypothesized to be at least partially offset by the circadian rhythm disruption associated with night shifts [31]. The impact of night shifts on communication is expected to be particularly impacted within dyads with divergent communication styles.

### Secondary outcomes

Sleep measures will be collected both leading up to and during the simulated shift work rotation. In the 7 days prior to simulation, participants wear an actigraphy device and complete a daily online sleep diary upon waking. During the simulated shift work rotation, all major sleep opportunities (>3 hours) are monitored via polysomnography.

Self-report measures are collected as part of the initial sleep and health survey as well as throughout the simulated shift work rotation. Timepoints and summaries of self-report measures are listed in Table 1. All self-report measures are collected via the Research Electronic Data Capture (REDCap) platform [45].

**Table 1:** Summary of self-report instruments by timepoint.

Self-report instruments by timepoint	Summary
*Initial Sleep and Health Survey*	
Morningness and Eveningness Questionnaire [46]	Measure of chronobiologic preference of morning or evening.
Insomnia Severity Index [47]	Measure for the detection of insomnia and related symptoms.
Berlin Questionnaire [48]	Screening tool for the detection of clinically relevant obstructive sleep apnea.
Shift Work Disorder Screening Questionnaire [49]	Screening tool for shift work disorder.
Ford Insomnia Response to Stress Test [50]	Measure of sensitivity of sleep disturbances in response to stress.
Beck Anxiety Inventory [51]	Measure of anxiety severity.
Patient Health Questionnaire-9 [52]	Measure of depression severity.
Copenhagen Burnout Inventory [53]	Measure of individual burnout, with subscales relating to personal, work-related, and client-related burnout.
Alcohol Use Disorders Identification Test – Consumption [54]	Measure of alcohol consumption habits.
Posttraumatic Stress Disorder (PTSD) Checklist for DSM-5 [55]	Measure of PTSD symptom severity, corresponds to *Diagnostic and Statistical Manual of Mental Disorders (5*^*th*^ *Edition)* criteria [56].
Adverse Childhood Experiences [57]	Measure of negative childhood experiences linked to negative health outcomes in adulthood.
EMS Chronic Stress Questionnaire [58]	Measure of work-related chronic stress, specific to EMS personnel.
EMS Critical Incident Inventory [59]	Measure of critical incident exposure and resultant stress symptoms specific to EMS personnel and work.
*7 Days At-home Sleep Monitoring*	
Sleep Diary [60]	Measure of sleep characteristics that allows for analysis of sleep efficiency.
*Simulated Rotation, Pre-Scenario*	
Samn-Perilli Fatigue Scale [61]	Measure for assessment of subjective fatigue.
Karolinska Sleepiness Scale [62]	Measure for assessment of subjective sleepiness.
*Simulated Rotation, Post-Scenario*	
NASA Task Load Index [63]	Measure of perceived workload.
*Simulated Rotation, Post-Decision Making and Cognitive Battery*	
Subjective assessment	Subjective assessment of workload and effort.
*Simulated Rotation, At the End of Each Shift*	
EMT-Teamwork Survey [64]	Measure of teamwork within small team EMS work environments.

EMS =  Emergency Medical Services; NASA =  National Aeronautics and Space Administration; EMT =  Emergency Medical Technician.

### Status and timeline

Recruitment began on October 17, 2023, and will continue until data collection is completed, which is estimated to be June 30, 2025. Data collection began on March 26, 2024. Final results are not expected to be available until 2026.

### Statistical analysis

Descriptive statistics of participant demographic information, pre-simulation sleep and health survey results, and secondary outcomes will be calculated to provide information about the participant group. Where relevant, t-tests and non-parametric equivalents will be used to determine statistically significant differences in these variables.

Overall GRS scores of work performance will be corrected for individual scenario difficulty to allow for linear mixed model (LMM) analysis of the within-participant impact of consecutive shifts (e.g., night shift 1 vs night shift 2), and within-shift impacts (e.g., first scenario as attendant vs second scenario as attendant). Changes in decision making strategy will be analysed through LMM comparison of Bayesian accuracy within timepoint (e.g., standard variant vs heuristic variant vs emotional content variant), within-shift (e.g., standard variant at timepoint 1 vs standard variant at timepoint 2), and between shifts (e.g., day shift 1 vs day shift 2). Risk preference will be examined through LMM analysis of within-shift and between-shift comparison of framing effect and BART derived risk-taking scores.

Individual profiles of biological marker results will be analysed using LMM to compare within-shift and between-shift changes for individual participants. Finally, quantitative measures of communication from openSMILE and LIWC outputs will be analyzed with LMM to compare changes in communication both within shift and between shift. A random effects intercept will be included in all models. All statistical analysis will be completed in R (version 4.4.0) and statistical code will be available on request.

### Data management plan

A designated space for all study data has been set up on the Monash University servers. This drive allows for secure long-term storage with controlled access. All scenario audio and video data, cognitive and decision making battery results, heart rate recordings, biological sample results, actigraphy, and polysomnography are stored on the shared drive. Sleep diaries, self-report survey data, pre-screen data, and consent forms are collected electronically via the REDCap platform hosted at Monash University.

### Dissemination

In recognition of the time required to complete this study, all participants will receive individualized summary reports about their specific performance. Dissemination of the full study results will be via peer reviewed publications and national and international conference presentations. Communication of paramedic profession specific results will take place via social media and through collaboration with paramedicine organizations and professional bodies, including those who assisted in recruitment.

## Discussion

The nature of paramedic work involves responding to unknown situations with limited resources at any time of the day. In these high-pressure and uncontrolled environments, paramedics make complex decisions, often while experiencing the impacts of sleep loss and circadian rhythm disruption due to shift work [1,65]. Shift work and sleep loss are known to adversely impact decision making and workplace performance [10]. In paramedics, however, limited research has examined how shift work influences paramedics’ decision making, stress physiology and work performance, which can have a significant impact on patients, their families, and the paramedics themselves. The present study uses a novel multi-day simulated shift rotation to explore the impact of shift work on paramedics’ work performance, decision making, stress responses, and communication. Findings from this study will provide valuable insights into how clinicians work and perform in response to simulated shift work conditions, which in turn, will inform strategies designed to promote occupational performance and reduce the risks associated with the demands of paramedic shift work.

Despite the novelty of this study protocol and the potential findings it will contribute to the paramedic research landscape, there are some limitations to consider. Due to the sensitive and unpredictable nature of paramedic work, evaluation of real-world work performance is not possible. The simulated paramedic scenarios in the current study will mimic the main elements of real-world patient care and scene management, but do not capture the myriad small details and decisions constantly being made during real paramedic calls. For instance, participants will not be required to drive and navigate to the call locations and hospitals, nor will they have to navigate challenging domicile entryways with their equipment. Moreover, the patients in the scenarios are not experiencing true medical emergencies, reducing the potential stress and urgency of participant actions. Despite best efforts to ensure all paramedic skills and techniques are fully enacted in the current study, there will be some actions that cannot be completed as they would be in the real world (e.g., placing a supraglottic airway). Additionally, as the simulations are completed with healthy research assistants in a single room with limited variations for set ups and furniture, it is safe to assume any manual handling of scenario patients will be more straightforward than what would be experienced in the real world. Finally, while all attempts are made to capture the stress and fatigue resulting from delays in hospital offload, there is limited ability to replicate the environmental conditions associated with a busy emergency department in the current study.

Despite the limitations associated with simulated paramedic scenarios, high fidelity simulations, such as those used in the current study, are well documented as a valid way to measure competence and improve performance in healthcare [66]. Both paramedic students and working paramedics routinely engage in SBA as a method for establishing and maintaining their clinical skills [17]. In an important step forward for this area, the present study uses a combination of state-of-the-art simulation facilities and validated decision-making tasks to examine the effects of a multi-day shift work rotation on paramedic performance. The simulated rotation allows for insights beyond those available from survey data alone. This includes information not just about paramedic decision making and work performance, but the impact of shift work rotations on physiological stress responses and communication. Through these measures, we will learn about the physiological changes paramedics experience as they work through a multi-day shift work rotation. These changes will be compared to work performance and decision making to form a holistic view of the impacts of shift work on paramedics.
